# Protective effects of *cornus mas* extract on *in vitro* fertilization potential in methotrexate treated male mice

**Published:** 2015-03-15

**Authors:** Leila Zarei, Rasoul Shahrooz, Rajabali Sadrkhanlou, Hassan Malekinejad, Abbas Ahmadi, Zahra Bakhtiary

**Affiliations:** 1 *Solid Tumor Research Center, Urmia University of Medical Science, Urmia, Iran; *; 2 *Department of Basic Sciences, Faculty of Veterinary Medicine, Urmia University, Urmia, Iran.*

**Keywords:** Cornus mas, *In vitro* fertilization, Methotrexate, Mice, Sperm quality

## Abstract

Current study was aimed to evaluating protective effects of *cornus mas* fruit extract (CMFE) in mice treated with methotrexate (MTX). For this purpose, 48 young mature male mice were divided into 6 groups. Control group received only normal saline (0.1 mL per day, intraperitoneally), and the second group was administered MTX (20 mg kg^-1 ^per week, intraperitoneally). The third, fourth and fifth groups received MTX daily oral doses of 250, 500 and 1000 mg kg^-1 ^CMFE as well as MTX. The sixth group was only given CMFE with a dose of 1000 mg kg^-1 ^per day, orally, for 35 days. Then, the animals were anesthetically euthanized and the sperms were separated from epididymis. DNA damage level, the amount of malondialdehyde (MDA) as well as *in vitro* fertility was evaluated. The number of sperms with damaged DNA and MDA level in MTX-treated group showed a significant increase compared to control group (*p* < 0.05). In groups receiving CMFE along with MTX, DNA damage level and MDA amount suggested a decrease in comparison with MTX group (*p* < 0.05). Also, *in vitro* fertilization and embryonic development in MTX-treated group was significantly lower than the control group, and the level of embryonic arresting was higher (*p* < 0.05). In groups which received CMFE along with MTX, *in vitro* fertility and embryonic development was higher than MTX group (*p* < 0.05) and the arrested embryos showed a decrease. This study suggested that *cornus mas* is able to ameliorate the side effects of MTX.

## Introduction

Infertility is a problem of many couples which causes psychological tension between partners and exerts a lot of stress on infertile couples.^1^ According to current statistics 15% of couples have infertility problems and in 50% of cases the cause of infertility originates from men solely or man and woman. In about 10% of cases, the main cause is the lack of sperm in semen.^2^

There are lots of known and unknown causes for infertility. One of the most common sources of infertility is chemotherapy.^4^ As a folate antagonist, methotrexate (MTX) was introduced in 1950 as a chemotherapeutic agent.^3^ Previous studies have shown that men treated with MTX, developed azoospermia and infertility. Methotrexate is used for a wide range of malignancies including acute and chronic leukemia, lymphoma, bladder cancer, breast cancer, and testicular tumor.^5^^,^^6^ Other applications of this medication include immunosuppression, treating the autoimmune disorders like arthritis rheumatoid and psoriasis. It has also immunosuppressive characteristic.^7^^,^^8^ Exposure to MTX results in severe decline in the number of sperms, and enhances the sperm's morphological disorders, testicular damage, DNA damage, and cellular sperm disorders.^9^ Also, toxicity of methotrexate has been reported in the other organs.^10^ Although, the exact and complete mechanism, which the MTX exerts its patho-logical impact remained unknown, several studies showed that MTX enhances the intracellular reactive oxygen species (ROS) generation.^11^^,^^12^ Therefore, the oxidative stress is considered as main side effect of MTX.^13^ Oxidative stress is an imbalance between ROS production and mechanisms restraining ROS.^14^^,^^15^ Because of multi-double bonded unsaturated fatty acids in plasma membrane and low preventive enzymes content, the sperms are highly susceptible against oxidative stress.^15^^,^^16^

*Cornus mas* (CM) is a member of Cornacea species. In traditional medicine, CMFE has been used for treating inflammation.^17^ Chemical analysis of CMFE shows that it is a rich resource of anti-oxidant and phenolic compounds. In addition, it contains vitamin C, B_1_, B_2_, E, and anthocyanins, flavonoids, and very high levels of oxalic acid.^18^^,^^19^

The object of current study was to investigate protective effects of CM hydro-alcoholic extract on MTX-induced derangements at sperm level and fertility potential in mice. 

## Materials and Methods

**Animals.** In this study forty-eight young (8 to 12 weeks) healthy male mice (NMRI strain) were used, and their fertility previously assessed by female mice. At first step the animals were kept for one week in standard conditions including temperature 22 ± 2 ˚C, humidity 30% to 60%, and with circadian illumination cycles of 14 hr light and 10 hr darkness. They had free access to food and water. 

**Preparations of hydro-alcoholic extract of** ***cornus mas*** **fruit. **In late summer, the CMS fruit was prepared from Qazvin province (Iran). The hydro-alcoholic extract was obtained using maceration method. After removing its stone the fruit has been dried in the air. Then, the powder was prepared and held in a closed chamber at 8 ˚C. Five hundred grams of powdered fruit was used to obtain extraction with a mixture of ethanol and water (7:3) at 25 ± 2 ˚C. The solution thoroughly held in 50 ˚C by vacuum apparatus for evaporating and its extract was kept in dry and freeze conditions in vacuum chamber until administration.^18^

**Animals and treatment groups. **After one week of environmental consistency and after weighing, male mice were randomly divided into six groups of including eight mice in each group.

Control group: Animals were considered as healthy and received normal saline (0.1 mL per day, intraperitoneally). 

Control sham, MTX group: Animals received 20 mg kg^-1 ^per week of MTX (Koçak Farma, Tekirdağ, Turkey), intraperitoneally.^18^

Experimental group 1 (MTX + CMFE): Animals were treated with CMFE (250 mg kg^-1 ^per day, orally) along with MTX.^19^^,^^20^

Experimental group 2 (MTX + CMFE): Animals were administered CMFE (500 mg kg^-1 ^per day, orally) along with MTX.^19^^,^^20^

Experimental group 3 (MTX + CMFE): *Cornus mas* extract (1000 mg kg^-1 ^per day, orally) was administered to the animals together with MTX.^19^^,^^20^

CMFE 1000 group (CMFE): Only *cornus mas* extract (1000 mg kg^-1 ^per day, orally) was administered to the animals.

The study period for all groups was 35 days. Then, the animals were anesthetically euthanized, with 25 mg kg^-1^ ketamine (Alfasan, Utrecht, The Netherlands) and the sperms were collected from epididymis.

**DNA strand damage level evaluation.** For evaluating sperms' DNA double strands, acridine orange staining was used. The percentage of spermatozoa stained was determined by counting 200 spermatozoa per case. The monomeric acridine orange, bound to normal double-stranded DNA shows a green fluorescence, while the aggregated acridine orange on single-stranded DNA produces a yellow to red fluorescence. After three times elution of sperm sample with PBS buffer, the precipitate final volume was set by PBS buffer. Smears of sperms from culture medium were prepared and after air-drying in the lab for 30 min transferred to a container having same ratios of acetone and ethanol. After that the slides dried in the air, they were placed in acridine orange solution for seven minutes and dried. Then, a 100 × fluorescent microscope (Model IX70; Olympus, Tokyo, Japan) was used for studying the slides and the results were reported as percentage.^21^^,^^22^

**Timing for injection of gonadotropins for** ***in vitro*** **fertilization (IVF). **After 35 days, mice were prepared for IVF. Ensuring the setup light/dark cycles for female mice necessary for sexual cycles and lasts two weeks at least, they were prepared for prompting of ovulation. This was done by intraperitoneal injecting 10 units PMSG hormone (Folligon; Intervet International BV, Boxmeer, The Netherlands) and after 48 hr intraperitoneal injecting 10 units HCG hormone (Folligon; Intervet International BV, Boxmeer, Holland) in 0.1 mL volume.

**Preparation of IVF culture medium. **A day before fertilization, the culture media necessary for fertilization were prepared and in order to reach stability were incubated at 37 ˚C under 5% CO_2_ for 12 hr. Fertilization dishes were dropped with Human Tubal Fluid (HTF; Sigma, St. Louis, USA) medium supplemented. One droplet of 500 µL was placed in each dish for fertilization and several 100 µL droplets were located in dishes for elution. All droplets were covered with mineral oil.

**Collecting of oocytes and IVF. **Approximately 13 hr after HCG injection (next morning), the female mice were euthanized. After shaving and sterilizing the abdominal area and laparotomy, the fallopian tubes were detached and placed in culture medium stabilized at 37 ˚C. Then, using "dissecting" method the oocytes were removed and after washing were added to droplets under mineral oil in HTF-BSA medium. Then, motivated and capacitated sperms with the concentration of 1 × 10^6^ total sperm per ml of culture medium were added. Fertilization process was recognized 3 to 5 hr after adding sperms observing two pronuclei. Therefore, fertilized oocytes (zygotes) were cultured in 100ml droplets under mineral oil for 120 hr.^23^ About 24 hr after the zygotes culture, the numbers of two cell embryos were counted and after 120 hr development of blastocysts and arrested embryos were evaluated under invert microscopy (Model IX70; Olympus, Tokyo, Japan).

The types of arrested embryos according to their fragmentation and necrosis^24^ were as follows:

Type I: Fully cellular lysing, necrotic and/or fragmented embryos.

Type II: Embryos with partially fragmented blastomeres.

Type III: Embryos with some fragmented blastomeres and/or cytoplasmic vesicles.

**Malondialdehyde (MDA) assaying. **To determine the lipid peroxidation rate, MDA content from the collected testis samples was measured using thiobarbituric acid (TBA) reaction as described previously. In short, 0.3 to 0.4 g of the testis samples were homogenized in ice-cold KCL (150 mM), and then the mixture was centrifuged at 3000 *g* for 10 min; 0.5 mL of the supernatant was mixed with 3 mL phosphoric acid (1% v/v) and then following vortex mixing, 2 mL of 6.7 g L^-1^ TBA was added to the samples. The samples were heated at 100 ˚C for 45 min, chilled on ice.

Finally, 3 mL N-butanol was added and the samples were further centrifuged at 3000 *g* for 10 min. The absorbance of supernatant was measured spectrophoto-meterically (Pharmacia LKB Biochrom Ltd., Cambridge, UK) at 532 nm and the MDA concentration calculated according to the simultaneously prepared calibration curves using MDA standards. The amount of MDA was expressed as nmol per mg protein of the samples.^25^ The protein content of the sample was measured according to the Lowry method.^26^

**Statistical analyses. **The data were analyzed by SPSS (Version 20; SPSS Inc., Chicago, USA) and one way ANOVA and Bonferroni test were used and IVF data were analyzed by Minitab (Version 16; Minitab, State College, USA). A *p*-value less than 0.05 was considered significant.

## Results

**Fig. 1:**
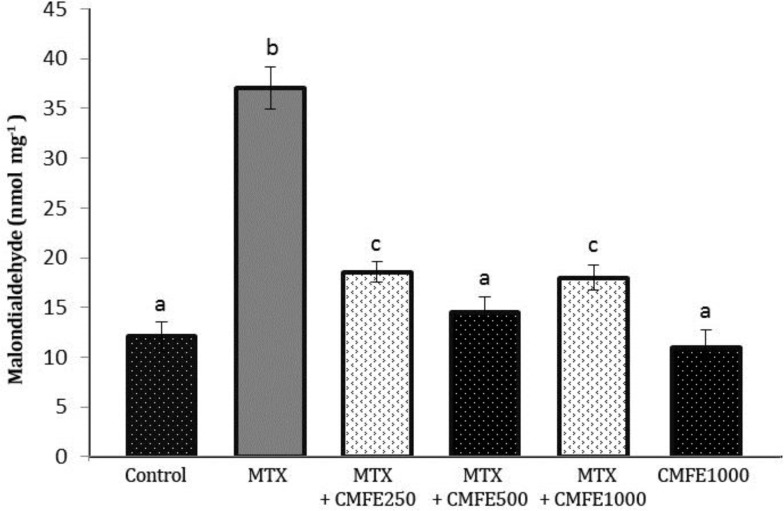
Comparison of testicular MDA levels in different groups.

^abc^ Different letters indicate significant difference between groups (*p* < 0.05).

**Malondialdehyde changes in testicular tissue. **Results from MDA assaying suggested that in MTX group the average levels of MDA (37.02 ± 2.12) in comparison with other groups increased significantly (*p* < 0.05). However, in group 3 and CMFE 1000 group no significant difference was observed in comparison to control group (Fig. 1).

**Fig. 2:**
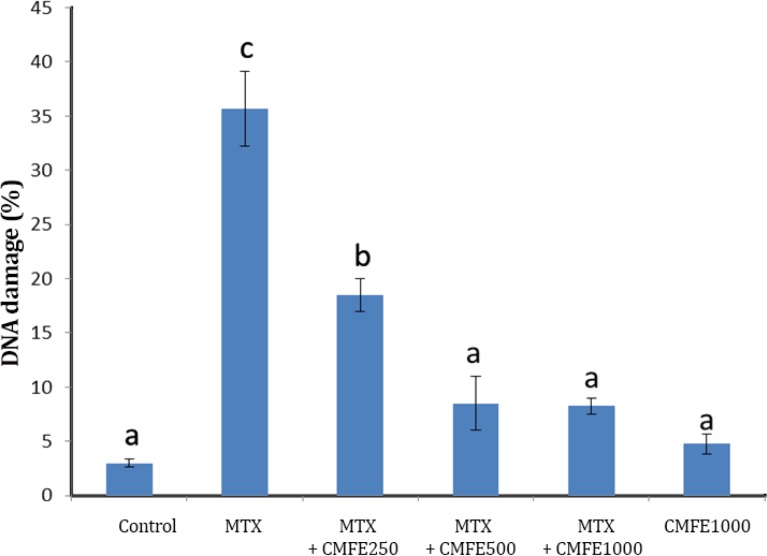
Effect of *cornus mas* administration in MTX induced reduction on the percentage of sperm DNA damage (Mean±S.E). ^a,b,c^ Different letters indicate significant differences between the experimental groups (*p* < 0.05).

**Fig. 3:**
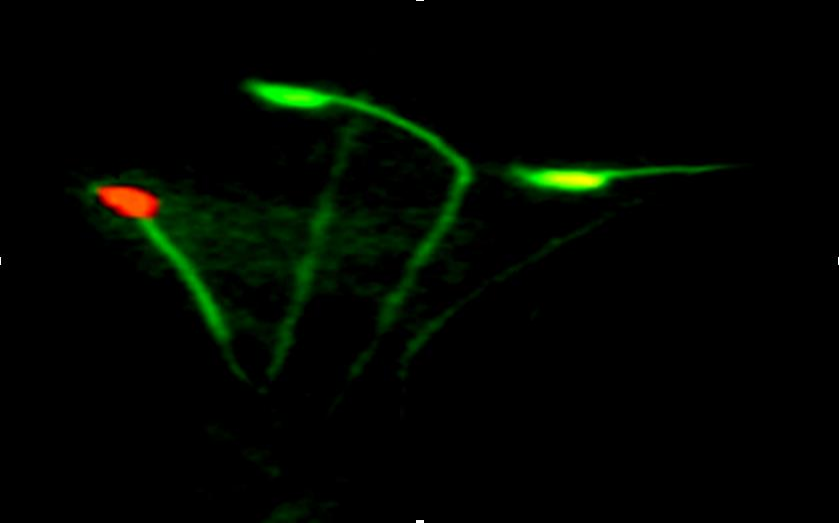
Micrograph of sperm. Green colored in head of sperm indicate DNA intact (a), Yellow to red colored indicate DNA damage in sperms (b), (Acridine orange staining, 1000×).

**DNA damage assessment. **The average percentage of sperms with damaged DNA was determined as follows: sperms with green color nucleus were considered as normal and sperms with orange to red nucleus were considered as DNA damaged ones according to damage level. In MTX group the average percentage of DNA damaged sperms (37.5 ± 3.44) showed a significant increased in comparison to all other groups (*p *< 0.05). However, the average percentage of sperms with damaged DNA in groups 2, 3 and CMFE 1000 groups were not significantly different with control group (Figs. 2 and 3). 

*In vitro* fertilization and embryonic development in different groups were presented in Table 1. Current findings suggested that percentage of fertilization in MTX group (77.49) was significantly lower than control group (92.64), (*p* < 0.05). In experimental groups 2 and 3, the percentage of fertilization non-significantly increased compared to control group. Whereas, CMFE 1000 group (95.12) showed significant difference in comparison to control group.

The percentage of two cell embryos producing, that indicate the beginning of cleavage in MTX group (73.87) showed a significant decrease comparing with control group (84.11), (*p *< 0.05). However, groups MTX + CMFE 250, MTX + CMFE 500 and CMFE + 1000 did not show significant difference. Percentage of two cell embryos in group CMFE + 1000 showed a significant decrease comparing with control group (*p* < 0.05).

Overall percentage of blastocyst in MTX group (42.34) suggested a significant decrease in comparison to control group (68.21), (*p* < 0.05). Only in MTX + CMFE 500 group and CMFE 1000 group were not observed significant difference in this parameter in comparison to control group. However, other groups have shown significant difference with control group (*p* < 0.05).

In MTX group, the total percentage (55.85) of arrested embryos in different development stages before blastocyst has increased in comparison with control group (*p* < 0.05). But, there was no significant difference between MTX + CMFE 500 and CMFE 1000 groups with control group. Other groups showed significant difference compared with control group (*p* < 0.05).

**Fig. 4:**
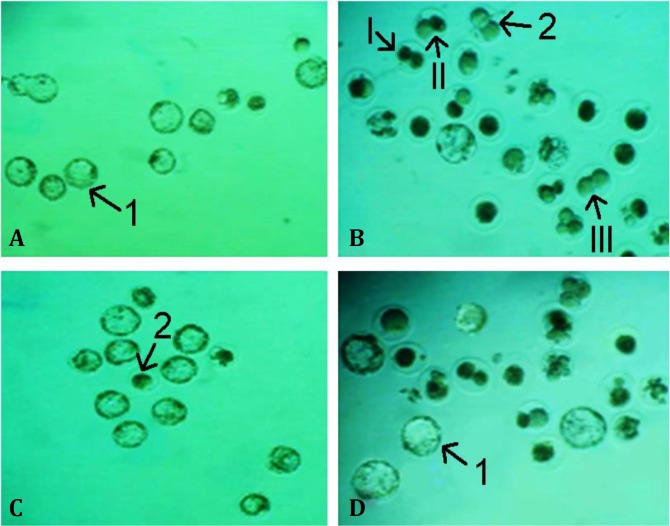
*In vitro* development of zygotes embryos at 120 hr after culture. **A)** Control group indicating most of embryos at blastocyst stage, **B)** MTX-treated group, **C)** Group received MTX with 500 mg kg^-1^ CMFE, and **D)** Group received MTX with 250 mg kg^-1^ CMFE, (400×). **1.** Blastocyst; **2.** Arrested zygote. Embryonic fragmentations: **I.** Type I embryo, **II.** Type II embryo, and **III.** Type III embryo.

Observation of the embryos developmental quality, considering fragmentation suggested a significant increase in the number of arrested embryos in MTX group compared with control group (*p* < 0.05). Most embryos were in type I category, while, CMFE with MTX could decrease the level of fragmentation of embryos in comparison with MTX group; resulting in that most of embryos were in type III category (*p* < 0.05), (Table 1, Fig. 4).

**Table 1:** Mean values (percentage) of fertilization and embryo development rate in different groups

**Groups**	**Oocytes**	**Fertilized oocytes**	**Two cell**	**Blastocysts**	**Arrested embryos**	**Type I Arrest**	**Type II Arrest**	**Type III Arrest**
**Control**	163	151 (92.64%)	127 (84.11%)	103 (68.21%)	48 (31.79%)	3 (1.98%)	7 (4.63%)	38 (25.16%)
**MTX**	149	111 (77.49%)^a^	82 (73.87%)^a^	47 (42.34%)^a^	64 (57.65%)^a^	49 (44.14%)^a^	10 (9.00%)	5 (4.50%)^a^
**MTX + CMFE 250**	137	115 (83.94%)^ab^	92 (80.00%)	53 (46.08%)^ac^	62 (53.91%)^ac^	14 (12.17%)abc	13 (11.30%)	35 (30.43%)^b^
**MTX + CMFE 500**	252	219 (86.90%)^b^	185 (85.25%)^b^	128 (58.44%)^b^	91 (41.50%)^b^	10 (4.60%)^b^	16 (7.30%)	65 (29.68%)^b^
**MTX + CMFE 1000**	152	136 (89.47%)^b^	96 (70.59%)^ac^	57 (41.91%)^ac^	79 (58.08%)^ac^	17a (12.50%)^bc^	10 (7.35%)	52 (38.23%)^ab^
**CMFE 1000**	164	156 (95.12%)bcd	141 (90.38%)bde	107 (68.58%)bcde	49 (31.41%)bcde	4 (2.56%) bde	11 (7.05%)	34 (21.79%)bce

**abcde:** Different superscripts indicate significant differences in each column (*p *< 0.05).

## Discussion

Most chemotherapeutics used for cancer have toxic side effects on different body systems.^27^ According to previous findings; testicular toxicity is reported as an important side effect for MTX.^12^^,^^13^ There is an increasing attention to protect the gonadal cells against chemo-therapeutic chemicals. In present study the protective effect of CMFE on MTX-induced derangements was examined. The number of sperms with damaged DNA and MDA level in MTX-treated group showed a significant increase compared to control group (*p* < 0.05), whereas in groups that received CMFE along with MTX, DNA damage level and MDA amount showed a decrease in comparison with MTX group (*p* < 0.05). *In vitro* fertilization and embryonic development in MTX-treated group was significantly lower than the control group, and the level of embryonic arresting was higher (*p* < 0.05). In groups received CMFE, *in vitro* fertility and embryonic development was higher than MTX group (*p* < 0.05) and the arrested embryos showed a decrease. This study suggested that *cornus mas* is able to ameliorate the side effects of MTX. Oxidative stress occurs following an imbalance between reactive oxygen species (ROS) and the antioxidant reserve system. ROS are produced as a result of normal cellular metabolism. Sperm, as the end production of testicular function, also produce free radicals of oxygen. Sperm capacitation, acrosome reaction, and sperm binding to the zona pellucida take place in association with low levels production of reactive oxygen radicals.^28^ On the other hand, enormous production of ROS leads to abnormality and infertility of sperms. Sperm membrane is rich in polyunsaturated fatty acids. This leads to membrane lipid peroxidation due to ROS increase.^29^ Therefore; peroxidative damage is a major factor in impaired sperm function.^30^

In the present study, MTX elevated the MDA content, which shows the MTX-induced lipid peroxidation. This finding was in accordance with previous reports.^31^^-^^33^ Antioxidative defense mechanisms in testis are important for protecting sperms against ROS. Previous studies reported that resveratrol, an antioxidant, lowers the MTX-induced lipid peroxidation.^34^^,^^35^ In correlation with this confirms the results of current study that in groups receiving MTX + CMFE the levels of MDA has been decreased significantly (*p* < 0.05). An increase in free radicals leads to oxidative damage, increasing lipid peroxidation, and finally DNA and protein damage.^36^ Oxidative stress has been reported to play an important role in the pathogenesis of MTX-induced testicular damage.^37^ The findings of staining by acridine orange to confirm other’s results suggested that MTX causes DNA damage and increases oxidative stress. Therefore, in groups treated with MTX, DNA damage has been increased significantly in comparison to groups receiving MTX + CMFE (*p* < 0.05).

Also, in the current study the levels of fertilization, and embryonic development in the mice treated with MTX was lower than control group and other groups receiving MTX + CMFE. Studies showed that there was positive correlation between sperm quality and viability and embryonic development in both *in vivo* and *in vitro*.^38^ This study also showed a high correlation between sperm quality and IVF results, and administration of CMFE along with MTX significantly increased the rate of fertilized oocyte, two cell zygotes and blastocysts, and decreased the percentage of arrested embryos. All of these effects of CMFE were dose dependent, and the optimum results obtained from 500 mg kg^-1^ CMFE. However, all factors causing defect in sperm quality and producing pathologic changes in it, will affect embryonic development. There are some evidences that MTX through high levels of ROS in oxidative stress causes azoospermia, decrease in the number of sperms, disorders in the morphology of sperms, sperm’s DNA,^39^ membrane and mitochondrial damage.^25^ All these cases will effect on sperm quality. Due to this reason, fertilization and viability of embryos declined in group treated with MTX. Another study confirmed the antioxidant role of CMFE and indicated a decrease in sperm damage and apoptosis as well as improvement in implantation and pregnancy rate.^40^ Another report on the role of antioxidants are confirmed the decreasing of damage in sperm and apoptosis, accordingly DNA, implantation and pregnancy rate is improved.^40^ Regarding to positive effects of CMFE on sperm parameters and interrelation of these parameters with successfully fertilization, the percentages of fertilized oocytes and blastocysts were raised and increasing of blocked embryos restricted in CMFE + MTX group.

In conclusion, this study showed that the stress oxidative is a major side effect of MTX that induced testicular damage and lowered sperm quality. However, the administration of CMFE along with MTX reduced oxidative stress and protected spermatogenesis and accordingly increased the rate of *in vitro* fertilization and decreased the embryonic arrest and embryonic fragmentation percentage. The results of present study suggest that these protective effects of CMFE may be due to its antioxidant property. 
